# Development of a Quantitative Method for Detection of Multiclass Veterinary Drugs in Feed Using Modified QuPPe Extraction and LC–MS/MS

**DOI:** 10.3390/molecules27144483

**Published:** 2022-07-13

**Authors:** Sunyeong Jang, Hyungju Seo, Hojin Kim, Hyoyoung Kim, Jongsung Ahn, Hyunjeong Cho, Sunghie Hong, Seunghwa Lee, Taewoong Na

**Affiliations:** 1 Experiment Research Institute, National Agricultural Products Quality Management Service, Yongjeon-ro 141, Gimcheon-si 39660, Korea; wts1424@naver.com (S.J.); hlhl103@naver.com (H.S.); rex7878@korea.kr (H.K.); hyo02@korea.kr (H.K.); hjcho201@korea.kr (H.C.); cybergus@korea.kr (S.H.); 2 National Agricultural Products Quality Management Service, Cheonnyeon-ro 1430, Yeonggwang-eup, Yeonggwang-gun 57049, Korea; j.ahn@korea.kr

**Keywords:** feed, veterinary drugs, QuPPe based, LC–MS/MS

## Abstract

A method was developed for the rapid and quantitative analysis of 30 veterinary drugs belonging to 17 classes (amphenicols (1), anthelmintics (1), cephalosporins (4), coccidiostats (1), lincosamides (1), macrolide (1), nitroimidazole (1), penicillins (3), phenylhydrazines (1), polypeptides (1), pyrethrins (1), quinolones (5), sulfonamides (3), tetracycline (3), neuroleptic agents (1), triazene trypanocidal agents (1), other. (1)) in feeds. The proposed method with a modified Quick Polar Pesticides (QuPPe) sample preparation was validated for the determination of 30 veterinary drugs in feed samples by liquid chromatography triple-quadrupole mass spectrometry (LC–MS/MS). The sample was extracted with methanol containing 1% acetic acid and purified by dispersive solid-phase extraction (d-SPE) with C18. Good linearity (*r*^2^ ≥ 0.98) was observed, and the LOQ values ranged from 10 to 200 µg/kg. Average recoveries ranged from 70.8 to 118.4%, and the relative standard deviation was ≤ 18.7%. This validated method was used in the determination of 30 veterinary drugs in 142 feed samples obtained from South Korea. The results show that lincomycin was present in only one of the tested feed samples, although it was detected at a value lower than the LOQ. In conclusion, this multi-residue method can be used for screening through the detection and quantitation of residual multiclass veterinary drugs in feed samples.

## 1. Introduction

Veterinary drugs are applied in feed industries worldwide. Generally, veterinary drugs are used to treat diseases that may affect livestock and promote animal growth [1]. However, the excessive use of these compounds can affect the food chain and, ultimately, humans due to the presence of residues in the final products [2,3]. These potential hazards can influence the occurrence of allergies, the mutagenesis of antibiotic-resistant bacteria, and health problems in the human system, such as through the pathogenic acquisition of drug resistance and carcinogenic effects due to chronic toxicity [1,4]. When livestock consume feed containing veterinary medicinal products, some may remain and accumulate in the animal’s tissues or organs. Additionally in this process, there is a possibility that new metabolites as well as the parent compound of veterinary drugs are generated and exist. Because their chemical structure is similar to that of drugs for the human body, exposure to the human body may result in problems, such as the disturbance of body homeostasis, specific drug reactions, and the inhibition of therapeutic effects due to the introduction of unnecessary therapeutic agents [5,6,7]. Therefore, it is important to establish and comply with the maximum residue levels (MRLs) of drugs in agricultural production and to manage the residue levels of these toxic substances [8].

The analysis of multiclass veterinary drugs of various polarities in feed is considered challenging because of the complexity and diversity of the matrix of feeds that often contain water, grains, milled products, minerals, vitamins, and fats [9]. Consequently, the complex composition of the feed often hinders the separation of veterinary drug residues by contributing co-extractives, which further interfere with identification and quantification during extraction. This handicap can lead to serious problems in sample analysis, including through co-eluting with target compounds, or causing ionization inhibition from electrospray ionization (ESI) sources or isobaric interference in mass-spectrometry (MS) detection. In addition, some veterinary drugs usually have small molecular weights, and the low mass of fragments when analyzed by MS/MS makes it difficult to detect highly polar compounds within polar samples [10].

In the last few years, analysis methods for residual polar compounds, including veterinary drugs in food and feed, have been continuously studied. For example, the liquid chromatography–electrospray ionization–tandem mass spectrometry (LC–ESI–MS/MS), or a combination of hydrophilic interaction liquid chromatography (HILIC) with MS, can be used in the analysis and identification of compounds even at low concentration levels [4,11,12,13,14,15]. In addition, methods for extracting polar compounds, including veterinary drugs, continue to be studied [7,13]. With the introduction of mass spectrometry, the most recently used extraction method is the QuEChERS (Quick, Easy, Cheap, Effective, Rugged, and Safe) method. This analysis method was first introduced in 2003 by Anastassiades et al. This analysis method includes an extraction process with acetonitrile and a purification process using a dispersed solid phase. Since then, the analytical method has been widely used in various forms by modifying the extraction solvent, salt, and purification reagent according to the analysis target in many laboratories. However, this analysis method uses a relatively non-polar solvent as an extraction solvent, so there is a limit to analyzing some medium-polarity and polar animal drugs [16,17,18]. In 2008, scientists from the European Reference Laboratory for Single Residue Methods (EURL-SRM) introduced an evolved residue analysis method for high polar pesticides, called QuPPe [19]. This method allows for the simultaneous extraction of high polar pesticide residues and their metabolites from foods and plant origin matrices using acidified methanol. Up until now, the QuPPe method has been modified, with different versions for various feed and food matrices for the analysis of diverse polar pesticides [10,20,21,22,23]. However, methods for the multi-analysis of polar or mid-polar veterinary drugs in feed samples remain scarce.

In a previous study, we developed a multi-residue analytical method for 197 pesticides, 5 mycotoxins, and 56 veterinary drugs based on QuEChERS using LC and GC-MS/MS [24]. On the basis of QuEChERS, some relatively polar veterinary drugs showed poor extraction efficiency. Therefore, we aimed to develop and validate a simple and effective method for the simultaneous determination of multiclass veterinary drugs in feed samples. The samples were extracted using a modified QuPPe extraction/clean-up procedure and analyzed using selective and sensitive LC–MS/MS. Method validation was conducted according to the guidelines from the Ministry of Food and Drug Safety (MFDS) [25]. In addition, this validated method was applied to the monitoring of 30 veterinary drug residues in 142 animal feed samples (70 feed ingredients and 72 compound feeds) consumed in South Korea.

## 2. Results and Discussion

### 2.1. Optimization of Sample Preparation

#### 2.1.1. Moisture Content

**Figure 1:**
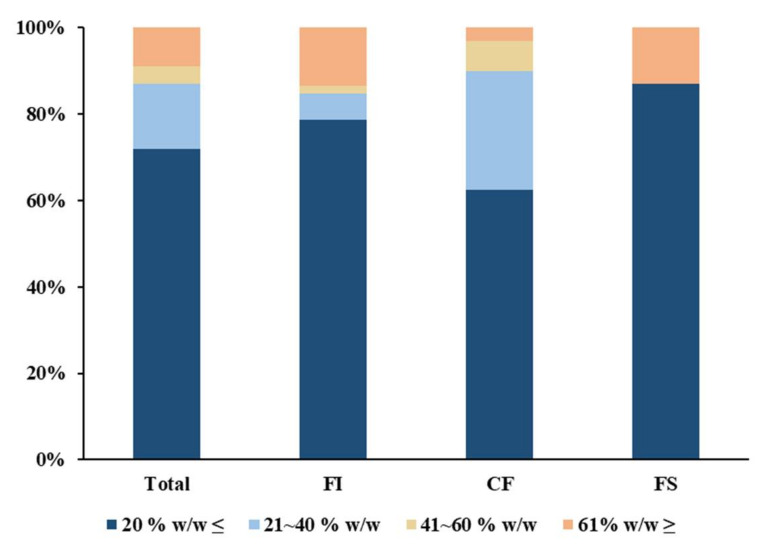
The moisture content of each feed to be investigated; 886 feeds in total: 476 feed ingredients (FIs), 379 compound feeds (CFs), and 31 feed supplements (FSs) (*n* = 3).

During extraction using a solvent such as methanol, the recovery varies depending on the moisture content of the sample. Accordingly, in the EURL QuPPe method, which is a pesticide analysis method for agricultural and livestock products, the analysis method was developed based on adjusting the sample such that it has a water content of 80% *w*/*w* or higher. In the case of general agricultural products and livestock products, the moisture content is relatively high, so a method involving correction of the moisture content, such as the EURL method, can be applied [19]. However, most of the feed is in a dry state. In the case of compounded feed, it is difficult to predict the moisture content because it is composed of various raw materials. Therefore, it is not possible to adjust the moisture content of the feed to 80% as in the existing QuPPe method. Accordingly, the actual moisture content of the distributed feed was checked. Monitoring the moisture content of 886 distributed feeds for about 5 years from 2017 to August 2021 revealed that 72% of the total surveyed feed had a moisture content of 20% *w*/*w* or less, and 87% of the total surveyed feed had a moisture content of less than 40% *w*/*w* (Figure 1). Looking at feeds with a moisture content of 40% *w*/*w* or more, most of the feed ingredients were leftover food feeds, the feed supplements were sugars, and the compound feeds were wet feeds and fiber feeds for ruminants.

**Figure 2:**
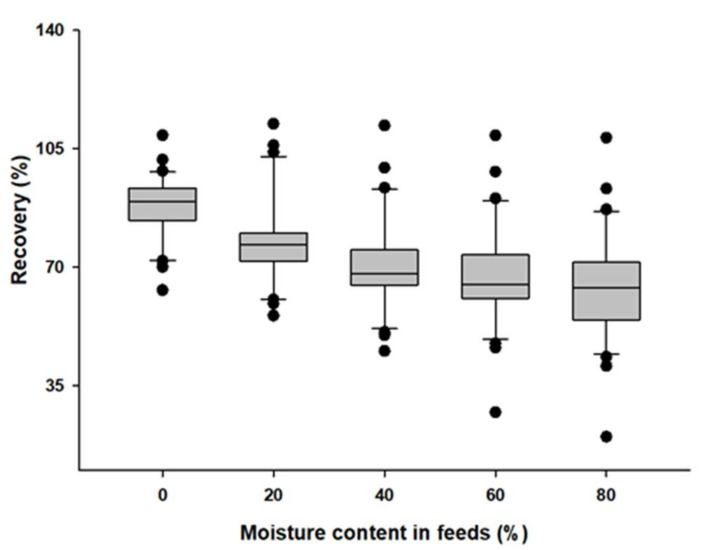
Effect of different moisture content in feeds on the recoveries of target compounds. All preparation was performed in triplicate using blank pet feed samples with moisture contents of 0, 20, 40, 60, and 80% *w*/*w* at middle validation levels (*n* = 3).

Therefore, in order to check the difference in recovery according to the moisture content of the feed during the extraction process, optimization was performed using blank pet feed samples with moisture contents of 0, 20, 40, 60, and 80% *w*/*w* at the middle validation levels (Table 1). As a result, the recovery where water was completely removed at 0% *w*/*w* was the highest, and most of the components showed a tendency toward decreased recovery as the feed water content increased (Figure 2). There was no significant difference in the recovery results between the feed containing 0 and 20% *w*/*w* water, but it was confirmed that the recovery of some components, such as phenothiazine, was slightly reduced. However, in feed with over 80% *w*/*w*, the recovery of most of the compounds decreased by over 20% *w*/*w*, and the recovery of some components, such as amprolium, decreased significantly. Comparing the recovery results by moisture content in the feed, as the moisture content increased, the median and average values of the recovery results tended to decrease, and the range widened. The recovery was confirmed to be relatively stable and satisfactory in the feed with 20% *w*/*w*. In addition, the recovery rate results in feed with a moisture content of 40% *w*/*w* also showed stable results. Therefore, the analysis method developed in this study can be applied to feeds excluding the supplementary feed, leftover food feed, and fiber feed for the ruminants mentioned above, and it was confirmed that quantitative analysis is possible regardless of the moisture content. We performed validation on the feed containing 20% *w/w*.

#### 2.1.2. Extraction Conditions

**Figure 3:**
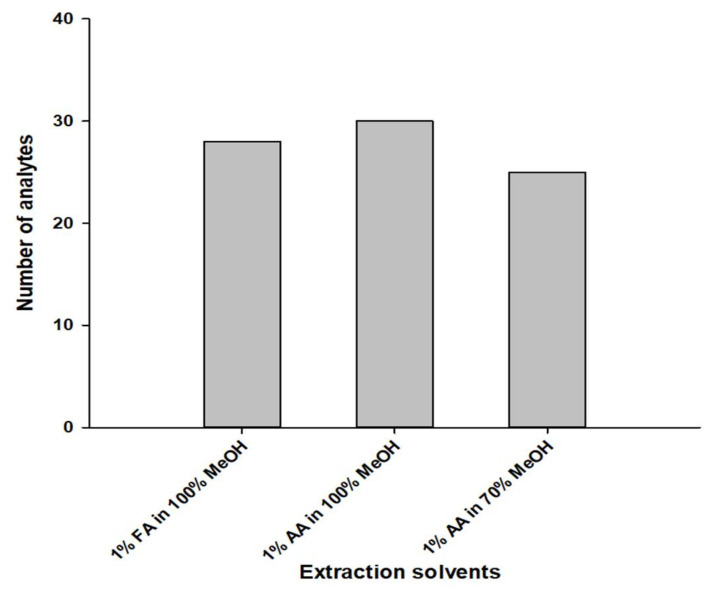
Effect of the different extraction solvents on the recoveries (70~120%) of target compounds at middle validation levels. These conditions included: (1) 1% formic acid (FA) in 100% methanol; (2) 1% acetic acid (AA) in 100% methanol; and (3) 1% AA in methanol/ethyl acetate (70:30, *v*/*v*). All preparations were performed in triplicate using blank pet feed samples (*n* = 3).

Methanol, acetone, acetonitrile, dichloromethane, and ethyl acetate are solvents commonly used for the extraction of various pesticide and veterinary drugs, of which methanol is the solvent most used to dissolve and extract polar and nonpolar chemicals. In many previous studies, methanol has been widely used in the extraction of veterinary drugs, such as tetracyclines, macrolides, and sulfonamide antibiotics [14,26,27,28,29,30]. Extraction conditions are mainly associated with the physical and chemical features of the target compounds as well as the features of the solvents. In this study, the selection of extraction conditions that were extensively applied to the analysis of the multiclass residues was an essential prerequisite. Thus, three sets of extraction conditions were designed for evaluating the influence of different solvents and the pH on the recoveries of the target compounds. These conditions were: (1) 1% formic acid in 100% methanol; (2) 1% acetic acid in 100% methanol; and (3) 1% acetic acid in methanol/ethyl acetate (70:30, *v*/*v*). The investigation was optimized for middle validation levels of the target compounds in a blank pet feed sample (Table 1). The results showed that, when 100% methanol was used as the extraction reagent, the recovery of target compounds ranged from 70 to 120% from 1% formic acid, whereas all target compounds in 1% acetic acid showed satisfactory recovery (Figure 3). Bacitracin and minocycline had recoveries ranging from 25 to 60% under 1% formic acid. Under the same condition of 1% acetic acid, the extraction efficiency was investigated using 100% methanol and methanol/ethyl acetate (70:30, *v*/*v*). When methanol/ethyl acetate (70:30, *v*/*v*) was used as the extraction reagent, less than 60% of bacitracin, cefalonium, and minocycline were recovered, whereas cefalexin and nafcillin showed higher than 120% recovery (Figure 3). Based on these results, the combination of 1% acetic acid in 100% methanol was selected as the optimal extraction conditions for further experiments. The accuracy and precision results for are summarized in Table S2.

### 2.2. Optimization of LC–MS/MS Conditions

The multiple reaction monitoring (MRM) mode is used by the developed analytical methods to quantify 30 veterinary drugs. It is possible to selectively quantify analytes inside complicated mixtures using the highly sensitive and specific mass-spectrometry technique known as MRM mode [31]. MRM was used to selectively detect and quantify veterinary drugs in feeds. Individual standard solutions were prepared and infused into the mass spectrometer to optimize the MRM parameters for each compound. Full scans in positive and negative electrospray ionization (ESI^+^ and ESI^−^, respectively) mode were conducted for the selection of suitable precursor ions. The results showed that almost all of the compounds produced abundant [M + H]^+^ ions in ESI^+^ mode. Different collision energies were used to determine which resulted in the most suitable product ions for MRM. After selecting the precursor ions, a product ion scan was performed to select three product ions. The most stable and sensitive ion was chosen for quantification, and the next most intensive ions were used for qualification. The LC–MS/MS conditions for each compound (retention time, precursor ion, product ion, and collision energy) were optimized to produce the ideal signals.

### 2.3. Method Validation

#### 2.3.1. Specificity, LOD, and LOQ

Good specificity means that the analytical method can differentiate between the target compound and other compounds or interfering substances [32]. When blank samples were analyzed, the specificity of the proposed method was satisfactory, as no interference peaks were detected in the ±2.5% retention time range for all target compounds. The LOD and LOQ values for veterinary drugs were defined as signal-to-noise (S/N) ratios of 3 and 10 as the lowest concentration levels of the spiked sample on a matrix-matched calibration (MMC) curve. This approach for calculating analytical limits is commonly used in mass spectrometry [14,26,28,33,34]. For each parameter, the most stringent values were chosen. The LOD and LOQ values of veterinary drugs ranged from 4 to 80 µg/kg and from 10 to 200 µg/kg, respectively (Table 1).

#### 2.3.2. Linearity

For linearity evaluation, seven standard solution concentrations ranging from 10 to 25,000 µg/kg in matrix extracts (corn, cow, and pet feed) were analyzed to determine the linearity. In general, the coefficient of determination (*r*^2^) was higher than 0.99 for most compounds in matrix extracts, indicating suitable linearity (Table 1). Bacitracin, danofloxacin, lincomycin, and oxytetracycline showed an *r*^2^ lower than 0.99. In particular, oxytetracycline showed *r*^2^ values of 0.9802, 0.9828, and 0.9837 in corn, cow, and pet feed, respectively. Weighted least-squares regression (WLSLR) with optimal weighting factors (1/x or 1/x^2^) can improve accuracy at lower concentration ranges [35].

#### 2.3.3. Matrix Effect

The matrix effect is one of the important factors influencing quantitative analysis using liquid chromatography–mass spectrometry. The matrix effect is caused by competition between the compound and co-eluting matrix compounds with ions formed at the LC–MS/MS interface. Responses can be negatively or positively influenced by the matrix effect. A matrix effect value of 0% means that no matrix effect has occurred. Negative values indicate the inhibition of the compound signal, and positive values indicate a compound signal induced by the matrix. Assessing the impact of matrix effects and using the appropriate methodology to attenuate or eliminate them can greatly increase the reliability of analytical methods. Therefore, effective evaluation of the matrix effect is required when establishing a method. A common evaluation method is to examine the ratio between the slope of the linear equation of the matrix calibration curve and the slope of the solvent calibration curve. A ratio between the two below 100% indicates that the matrix effect is mainly suppressed. A ratio greater than 100% indicates that the matrix primarily has an enhancement effect [36]. A series of standard solutions with concentrations from 10 to 25,000 µg/kg were prepared using solvent and matrix solutions. Linear regression was fitted to the peak area (y) corresponding to the determined retention time and concentration (x) of each compound. As a result, some of the tested compounds showed a strong matrix effect, and the slope ratios of the two curves in the three matrices (corn, cow, and pet feed) ranged from −99.21 to 99.89% (Table S1). About 60% of the total compounds had low or negligible matrix effects. In corn, cow, and pet feed, 22, 20, and 15 compounds, respectively, showed weak substrate effects in the <±50% range. However, in cow and pet feed, about 40% of the total ingredients showed a strong matrix effect in the >±50% range. All of the tested matrices showed a strong negative matrix effect on the detection of amprolium, cefalexin, and orbifloxacin. Additionally, a strong positive matrix effect was observed on diethylcarbamazine, diminazene, florfenicol amine, lincomycin, and marbofloxacin, ranging from 76.18 to 99.89% in pet feed (Table S1).

In a previous study, Kim et al. (2021) confirmed the matrix effect of 64 veterinary drugs in fish products. Similar to our results, a strong matrix effect of −90% or more was confirmed in more than 20 veterinary drugs [34]. Additionally, Chiaochan et al. (2010) confirmed the matrix effect of 24 veterinary drugs on chicken muscle [15]. A strong matrix effect of more than −90% was observed with amprolium, danofloxacin, and lincomycin. Danezis et al. (2016) confirmed the matrix effect of 28 pesticides, veterinary drugs, and mycotoxin on several foods, including apples, wheat, apricots, chickpeas, lettuce, milk, and onions [14]. Contrary to our results in which danofloxacin, doxycycline, and oxytetracycline showed a matrix effect of ±50% in feed, a strong matrix effect of about ±80% or more was observed in milk and meat. Based on these results, to reduce the impact of matrix effects on the analysis of target compounds, an MMC was used for quantification.

#### 2.3.4. Accuracy and Precision

Accuracy and precision are key parameters to be assessed for method validation. Accuracy is the degree of agreement of test results generated by the method to the true value, and it is measured by spiking the sample matrix of interest with a known concentration of compound standard and analyzing the sample using the method being validated [37]. Accuracy was demonstrated with five replicates, each in two different laboratories, by spiking three concentration levels in corn, cow, and pet feed, respectively. The calculations were performed using MMC standards to take into account the matrix effect in quantitation. According to the MFDS guidelines from South Korea, when the validation levels were 10–1000 and >1000 µg/kg, the recovery ranges were 70–120 and 70–110%, respectively [25]. According to the CODEX and SANTE guidelines, mean recoveries of 70–120% with relative standard deviation (RSD) is acceptable when referring to validation experiments of 20%, while in certain cases, typically with multi-residue methods, recoveries outside this range may be acceptable under certain conditions [38,39]. For compounds with recovery values lower than 70% or higher than 120%, the method is still able to serve as a quantitative method, but the final concentration of the compound in the sample has to be corrected for recovery [39]. Generally, most of the compounds presented acceptable recoveries in all matrices, ranging from 70% to 120%. Cefalonium showed low recoveries in most matrices. In particular, the high validation level (5000 µg/kg) of cefalonium had the lowest recovery in cow feed (70.79%) but satisfied the >70% level specified in MFDS guidelines. However, the average cefalonium recovery was slightly higher in cow feed (80.88%). Additionally, low average recoveries (<80%) were observed for lincomycin in corn feed, cefalonium in cow feed, and cefalexin in pet feed. By contrast, diethylcarbamazine showed high recoveries in most matrices, and the values did not exceed the MFDS guideline level of 120% [25]. However, the average diethylcarbamazine recovery was lower in corn feed (84.17%) compared with other matrices. In addition, high average recovery (>110%) was observed for monoacetyl dapsone in corn feed. Additionally, the average recoveries of all compounds were 99.15, 94.16, and 91.17% in corn, cow, and pet feed, respectively, which was found to be higher in the feed ingredients (corn) than in the compound feeds (cow and pet). Corn feed recoveries ranged from 81.56 to 111.82%, cow feed recoveries ranged from 80.88 to 104.45%, and pet feed recoveries ranged from 78.54 to 101.83%.

**Figure 4:**
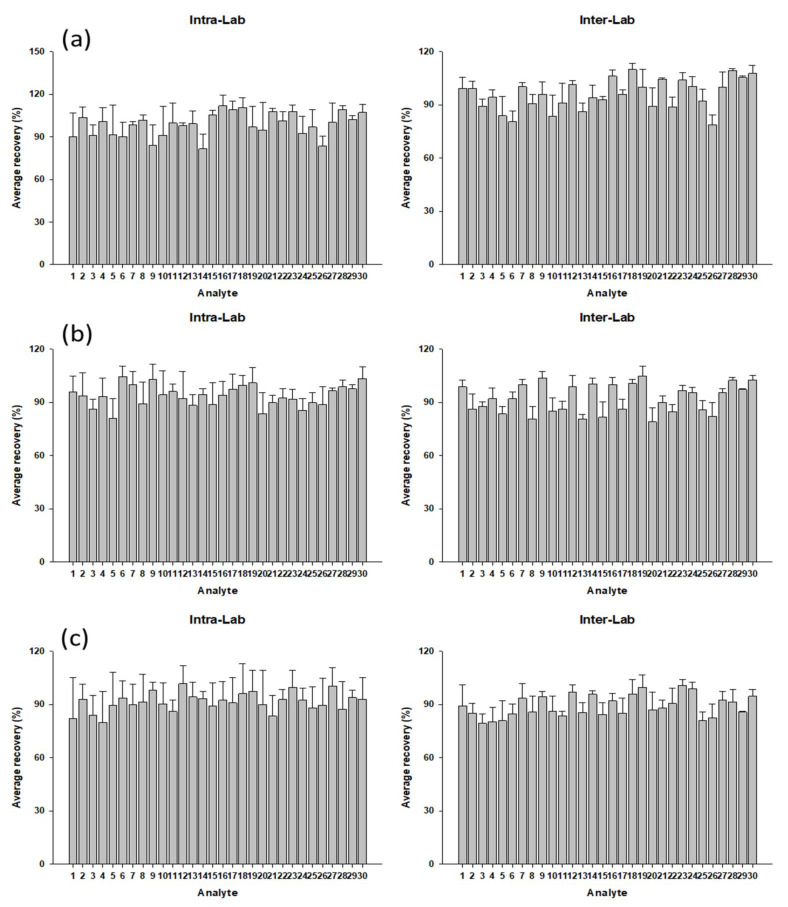
The average recoveries of 30 veterinary drugs intra-lab and inter-lab in (**a**) corn feed, (**b**) cow feed, and (**c**) pet feed (*n* = 5). Veterinary drug name: (1) amprolium; (2) bacitracin; (3) cefadroxil; (4) cefalexin; (5) cefalonium; (6) cephapirin; (7) cloxacillin; (8) danofloxacin; (9) diethylcarbamazine; (10) diminazene; (11) doxycycline; (12) florfenicol amine; (13) isometamidium; (14) lincomycin; (15) marbofloxacin; (16) metronidazole-OH; (17) minocycline; (18) monoacetyl dapsone; (19) nafcillin; (20) ofloxacin; (21) orbifloxacin; (22) oxytetracycline; (23) penicillin V; (24) phenothiazine; (25) phthalylsulfathiazole; (26) sarafloxacin; (27) succinylsulfathiazole; (28) sulfisoxazole; (29) tetramethrin; (30) tulathromycin.

Precision is the degree of agreement among individual test results when the procedure is repeatedly applied in multiple samplings, which is measured by injecting a series of standards or analyzing a series of samples from multiple samplings from a homogeneous lot. From the measured SD and mean values, precision is calculated as RSD [37]. Precision was demonstrated with five replicates each in two different laboratories by spiking three concentration levels in corn, cow, and pet feed, respectively. According to the MFDS guidelines from South Korea, when the validation levels are 10–100, 101–1000, and >1000 µg/kg, the intra-lab RSD values are 22, 18, and 14%, respectively [25]. The RSD values for most compounds were less than 20%, showing that the method has adequate precision (Table S1). The highest intra-lab RSD value at the high validation level (500 µg/kg) of doxycycline was measured in the pet feed at 17.30%, but it did not exceed the MFDS guideline level of 18%. According to the MFDS guidelines from South Korea, when the validation levels are 10–100, 101–1000, and >1000 µg/kg, the inter-lab RSD values are 34, 25, and 19%, respectively [25]. Inter-lab RSD values were lower than 20% for all analytes. The highest inter-lab RSD value at the middle validation level (50 µg/kg) of amprolium was measured in the corn feed at 27.19%, but it did not exceed the MFDS guideline level of 34% [25]. In conclusion, these results demonstrate the suitable accuracy and precision of the method. The accuracy and precision results for all concentration levels, compounds, and matrices are summarized in Figure 4 and Table S1.

In previous reports, there have been various studies analyzing veterinary drugs in fishery and livestock products. Kim et al. (2020) established a method to analyze 60 veterinary drugs in flatfish using LC–MS/MS [40]. In a similar study, Kim et al. (2021) also developed a method for the analysis of 64 veterinary drugs in fishery products using LC–MS/MS [34]. In addition, Yu et al. (2012) and Kanda et al. (2015) developed a method to analyze 15 and 37 veterinary drugs, respectively, in livestock products using LC–MS/MS [7,41]. These previous studies provided the quantification, screening, and quantification of various classes of veterinary drugs (benzimidazoles, cephalosporins, coccidiostats, macrolides, nitroimidazoles, penicillins, quinolones, quinoxalines, sulfonamides, and tetracyclines) in fisher products (flatfish, eel, shrimp, salmon, and red sea bream) and livestock products (swine, bovine, chicken, and prawn). However, there are few previous studies analyzing various classes of veterinary drugs in feed. Kim et al. (2016) developed a method to analyze 16 sulfonamides in animal feed using LC-UV [42]. Furthermore, in a similar study, Lopes et al. (2012) and Patyra et al. (2019) developed a method to analyze several sulfonamides in animal feed using LC-UV and LC–MS, respectively [1,43]. Considering that there are not many previous studies analyzing veterinary drugs in feed, these previous studies may be helpful, but they are limited to sulfonamide analysis. In this study, various classes of veterinary drugs, including sulfonamide, can be analyzed in feed, so this limitation is expected to be overcome.

### 2.4. Analysis of Real Feed Samples

The established method was applied in the determination of 30 veterinary drugs in 142 feed samples (70 feed ingredients and 72 compound feeds) obtained in several regions of South Korea. According to the Ministry of Agriculture, Food, and Rural Affairs (MAFRA) guidance from South Korea, the target veterinary drugs in feed should not be detected in feed [44]. The presence of a positive sample was confirmed by comparing the retention time and product ion ratio obtained with the calibration standards. Among the feed samples, only one feed ingredient sample was positive for lincomycin, although the concentration (9.60 μg/kg) or contamination level was below the LOQ. This method is very efficient for recovering extractable residues and shows good applicability. It is necessary to more strictly manage the contamination standards for compounds, such as veterinary drugs in domestic animal feeds. In addition, these results suggest the need for the continuous monitoring of veterinary drugs in feed ingredients and compound feeds.

## 3. Materials and Methods

### 3.1. Chemical and Reagents

All standards of veterinary drugs were purchased from AccuStandard Inc. (New Haven, CT, USA) and Chiron AS (Stiklestadveien 1, NO-7041 Trondheim, Norway). Acetonitrile (ACN), methanol (MeOH), ethyl acetate, and water were provided by Merck (Darmstadt, Germany). All the solvents used in the analysis were LC–MS grade. Purified water was generated by using a Milli-Q system (Millipore, Bedford, MA, USA). Octadecylsilane (C18) was provided by Biotage (Uppsala, Sweden). Ammonium formate and acetic acid were purchased from Sigma-Aldrich (St. Louis, MO, USA). Formic acid was supplied by Fisher Scientific (Pittsburgh, PA, USA). Individual stock solutions of pure standards from 100 to 1000 μg/mL were prepared in ACN or MeOH and stored at –20 °C. The working solutions were prepared by diluting each stock solution with ACN to the required concentration.

### 3.2. Optimization of Sample Preparation

The extraction procedure was based on the QuPPe method developed by EURL, and the extraction solvent was modified. In addition, a comparative experiment was performed on the moisture content [19]. Optimization studies were conducted by spiking analytes into blank pet feed obtained from feed factories and markets in South Korea. All feed samples were ground and stored in the freezer at –20 °C. In order to compare the extraction efficiency of analytes according to the moisture content of the feed and the type of extraction solvent. All comparative experiments were performed in triplicate at the middle validation level in a blank pet feed sample.

#### 3.2.1. Moisture Content

The effect of moisture content on the recovery of 30 veterinary drugs was assessed at five moisture levels (0, 20, 40, 60, and 80% *w*/*w*) using the pet feed. The range of the moisture content was chosen because 70% of the domestically distributed feed has a moisture content of 20% *w*/*w* or less, and the QuPPe technique proposed by EURL is based on samples with a moisture content of 80% *w*/*w* or more [19]. To determine the moisture content of a sample, first an aluminum weighing bottle is pre-dried and weighed to a constant. A weighing bottle containing 2 g of the sample is dried in a constant temperature dryer at 135 ± 2 °C for 2 h. The sample is dried to a constant weight at 110 °C and then cooled in a desiccator for 30 min. The moisture content (M) is then calculated using the formula:

M (%) = (W_b_ − W_a_)_/_S_w_ × 100

With W_b_ the weight of the weighing bottle containing the sample before drying; W_a_ being the dry weight of the weighing bottle containing the sample; and S_w_ being the sample weight.

#### 3.2.2. Extraction Solvent Conditions

The optimization of the extraction solvent was performed by comparing the recovery of 30 veterinary drugs using three different solvents containing 1% formic acid in MeOH, 1% acetic acid in MeOH, and 1% acetic acid in MeOH/ethyl acetate (70:30, *v*/*v*).

### 3.3. Sample Preparation Using the Optimized QuPPe Method

**Figure 5:**
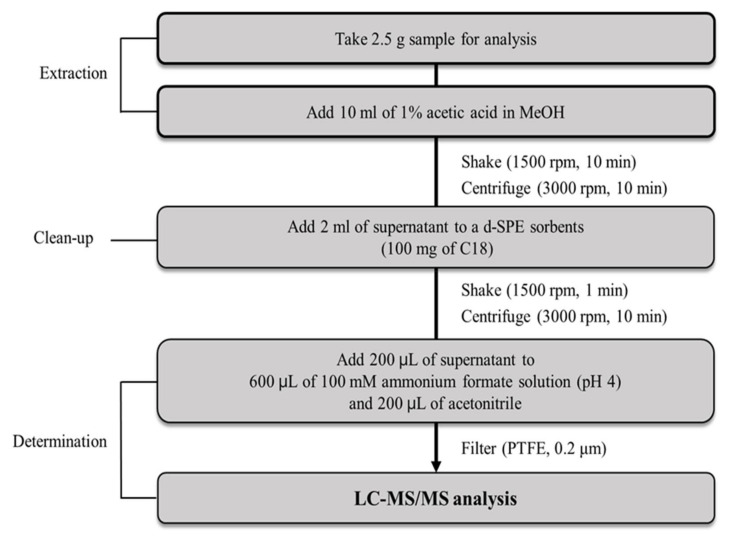
Analytical flow of the optimized method for the analysis of veterinary drugs in feed samples.

Blank samples were examined to confirm the absence of target veterinary drug residues and used to develop and validate the proposed method. About 2.5 g of the sample was weighed in a 50 mL polypropylene centrifuge tube. Next, 10 mL of methanol with 1% acetic acid was added. The mixture was shaken at 1500 rpm for 10 min. Extracted samples were centrifuged at 3000 rpm for 10 min. For sample clean-up, 2 mL of the supernatant obtained from centrifugation was added to a centrifuge tube containing 100 mg of C18 and shaken at 1500 rpm for 1 min. After being shaken, samples were centrifuged at 3000 rpm for 10 min. All the samples were filtered using a 0.2 μm PTFE syringe filter for LC–MS/MS analysis. A flow chart of the developed analysis method is shown in Figure 5.

### 3.4. LC–MS/MS Condition

All the veterinary drugs were analyzed on an AB SCIEX QTRAP 5500 triple-quadrupole mass spectrometer (AB Sciex, Toronto, Canada) equipped with a Shiseido Nanospace SI-2 liquid chromatography system (Shiseido, Kyoto, Japan). Chromatographic separation was carried out in an Imtakt Unison UK-C18 column (150 mm × 3.0 mm, 3.0 μm, 120 Å), and the column oven temperature was maintained at 40 °C. Water with 5 mM ammonium formate and 0.1% formic acid (A) and acetonitrile with 5 mM ammonium formate and 0.1% formic acid (B) were used as mobile phases.

**Figure 6:**
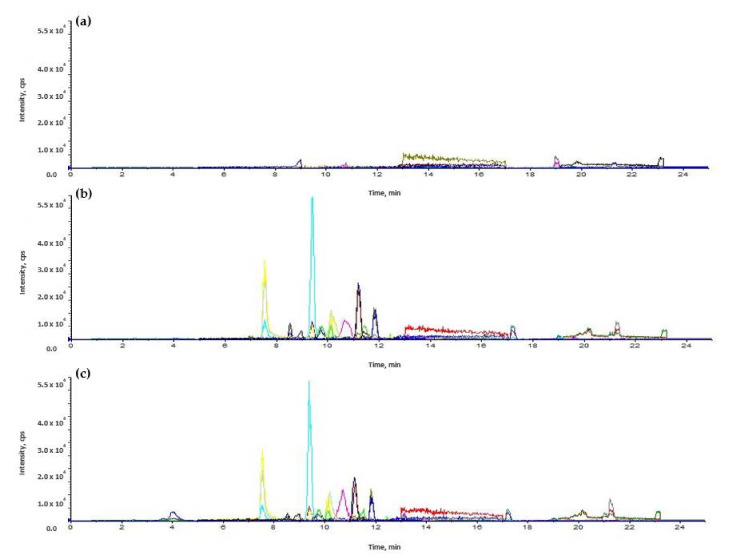
Representative multiple-reaction monitoring chromatograms of: (**a**) blank; (**b**) middle concentration standard; (**c**) middle concentration sample.

For high-performance liquid chromatography (HPLC) separation, a linear gradient program was used, with 5% B for 0–1.0 min, 5%–95% B for 1.0–18.0 min, 95% B for 18.0–22.0 min, 95–5% B for 22.0–22.1 min, and 5% B for 22.1–30.0 min. The total run time was 30.0 min, and the flow rate was 0.3 mL/min. The analysis was performed using electrospray ionization (ESI) in positive-ion mode at 4500 V or negative-ion mode at −4500 V. The instrument parameters were optimized as follows: interface temperature, 300 °C; heat block temperature, 400 °C; DL temperature, 250 °C; nebulizing gas flow, 3 L/min; heating gas flow, 10 L/min; and drying gas (nitrogen) flow, 10 L/min. To optimize the MRM of each veterinary drug, precursor ions were set by referring to the food code from MFDS, and the mixture of analytes was directly injected into the ESI source of the MS. Optimized LC–MS/MS conditions and representative chromatograms are described in Table 2 and Figure 6.

### 3.5. Method Validation

The proposed method was validated by spiking blank feed samples, including corn, pet, and cow feeds, according to the MFDS guidelines for validation and analytical quality control [25]. The following parameters were evaluated by assessing the linearity of the calibration curve, matrix effect, specificity, LOD, LOQ, accuracy (as % recovery), precision (as repeatability % RSDs), and reproducibility (as inter-lab and intra-lab).

In this study, MMC was used because of the complexity of the feed matrix. For the MMC curves, the calibration ranges of standards were evaluated from 10 to 25,000 µg/kg for each feed sample. The linearity of the calibration curve was appraised by *r*^2^, which was expected to be ≥0.98. The matrix effects were assessed by comparing the slopes of the calibration curves of different matrices and solvents. The LODs and LOQs were calculated from the lowest concentration of an analyte and showed S/N values of 3 and 10, respectively. To evaluate the recovery study, the blank feed matrices of the corn, pet, and cow feeds were fortified with the mixture of target compounds at 5 × LOQ, 10 × LOQ, and 25 × LOQ levels with five replicates [9]. The specificity of the method was determined by analyzing solvent and spiked samples. The absence of background peaks at the retention times of the target compounds showed that no interference occurred. Accuracy and precision (intra-lab and inter-lab) were obtained according to the criteria specified in the MFDS guidelines [25].

### 3.6. Real Feed Sample Analysis

For the monitoring of about 30 veterinary drugs, a total of 142 animal feed samples (70 feed ingredients and 72 compound feeds) were randomly obtained from feed factories and markets in various locations in South Korea.

### 3.7. Data Analysis

All analytical data were processed using Analyst software (AB SCIEX, version 1.7.1). The median values of experimental data were calculated using Microsoft Excel 2013 (Microsoft Co., Redmond, WA, USA). Statistical analysis of box-and-whisker plots was carried out with SigmaPlot 12.0 (Systat Software Inc., Erkrath, Germany).

## 4. Conclusions

In this study, a liquid chromatography–tandem mass spectrometry method based on QuPPe extraction was developed for the qualification and quantification of the residues of veterinary drugs in widely-used animal feeds. Thirty veterinary drugs showed quantitative limits ranging from 10 to 200 µg/kg and recoveries ranging from 70.04 to 119.94%. As the method complies with the requirements of the analytical quality control standards of the MAFRA guidelines from South Korea, the present method can be recommended for the regulatory testing of feeds for residues of veterinary drugs. This optimized method was applied to 142 samples of feed ingredients and compound feeds commonly consumed in South Korea. As a result, only one feed sample tested positive for lincomycin, although at a concentration below the LOQ. However, it is necessary to more strictly control the contamination standards for residual veterinary drugs in feed ingredients and compound feeds in South Korea. The developed multi-residue quantitative detection method is considered applicable for screening through the qualification and quantitation of residual veterinary drugs in feed ingredient and compound feeds.

## Figures and Tables

**Table 1:** Calibration data for optimization of the limit of detection (LOD), limit of quantification (LOQ), linear range, determination coefficient (*r*^2^), and spiked concentration for the recovery of 30 veterinary drugs in corn, cow, and pet feeds.

Analyte	logP	LOD (µg/kg)	LOQ (µg/kg)	Linear Range (µg/kg)	*r* ^2^			Spiked Concentration (µg/kg)		
					Corn Feed	Cow Feed	Pet Feed	Low	Middle	High
**Amphenicols (1)**										
Florfenicol amine	2.2	4	10	10–1250	0.9993	0.9999	0.9998	50	100	250
**Anthelmintics (1)**										
Diethylcarbamazine	0.57	4	10	10–1250	0.9966	0.9977	0.9975	50	100	250
**Cephalosporins (4)**										
Cefadroxil	1.18	80	200	200–25,000	0.9959	0.991	0.9906	1000	2000	5000
Cefalexin	1.47	80	200	200–25,000	0.9946	0.9995	0.9982	1000	2000	5000
Cefalonium	−0.19	80	200	200–25,000	0.9983	0.9908	0.998	1000	2000	5000
Cephaprin	1.25	8	20	20–2500	0.9952	0.9926	0.9988	100	200	500
**Coccidiostats (1)**										
Amprolium	−2.86	4	10	10–1250	0.9983	0.9985	0.9984	50	100	250
**Lincosamides (1)**										
Lincomycin	−0.53	4	10	10–1250	0.9993	0.9893	0.9852	50	100	250
**Macrolide (1)**										
Tulathromycin	2.65	8	20	20–2500	0.9953	0.9994	0.9985	100	200	500
**Nitroimidazole (1)**										
Metronidazole-OH	0.62	8	20	20–2500	0.9964	0.999	0.9928	100	200	500
**Penicillins (3)**										
Cloxacillin	2.88	4	10	10–1250	0.9984	0.9999	0.9908	50	100	250
Nafcillin	2.81	8	20	20–2500	0.9963	0.9983	0.9909	100	200	500
Penicillin V	2.09	8	20	20–2500	0.994	0.9874	0.9963	100	200	500
**Phenylhydrazines (1)**										
Diminazene	4.04	4	10	10–1250	0.9986	0.9985	0.9987	50	100	250
**Polypeptides (1)**										
Bacitracin	3.72	80	200	200–25,000	0.9866	0.9871	0.9957	1000	2000	5000
**Pyrethrins (1)**										
Tetramethrin	2.9	4	10	10–1250	0.9898	0.9969	0.997	50	100	250
**Quinolones (5)**										
Danofloxacin	2.07	4	10	10–1250	0.99	0.989	0.9857	50	100	250
Marbofloxacin	0.58	4	10	10–1250	0.9981	0.9913	0.9954	50	100	250
Ofloxacin	1.55	4	10	10–1250	0.999	0.9984	0.9912	50	100	250
Orbifloxacin	3.03	4	10	10–1250	0.9961	0.9963	0.9977	50	100	250
Sarafloxacin	2.77	8	20	20–2500	0.9992	0.9964	0.9975	100	200	500
**Sulfonamides (3)**										
Phthalylsulfathizole	4.12	8	20	20–2500	0.9966	0.9951	0.9984	100	200	500
Succinylsulfathiazole	0.87	8	20	20–2500	0.9943	0.999	0.9926	100	200	500
Sulfisoxazole	3.41	4	10	10–1250	0.9986	0.9998	0.9963	50	100	250
**Tetracycline (3)**										
Doxycycline	0.35	8	20	20–2500	0.9963	0.9996	0.9997	100	200	500
Minocycline	0.05	8	20	20–2500	0.9925	0.991	0.9914	100	200	500
Oxytetracycline	−0.54	8	20	20–2500	0.9802	0.9828	0.9837	100	200	500
**Neuroleptic agents (1)**										
Phenothiazine	3.21	4	10	10–1250	0.9911	0.9916	0.9877	50	100	250
**Triazene trypanocidal agents (1)**										
Isometamidium	4.4	4	10	10–1250	0.9979	0.9977	0.994	50	100	250
**Other (1)**										
Monoacetyl dapson	3.8	8	20	20–2500	0.9953	0.9981	0.9964	100	200	500

**Table 2:** Retention time (RT) and MS/MS conditions for each compound.

Compound Name	Chemical Group	RT (min)	ESI	Quantification, *m*/*z* (CE, eV)	Qualification 1, *m*/*z* (CE, eV)	Qualification 2, *m*/*z* (CE, eV)
Amprolium	Coccidiostats	2.8	+	243.09 > 150.20 (17)	243.09 > 94.10 (33)	243.09 > 81.20 (51)
Bacitracin	Polypeptides	15.3	+	474.90 > 199.10 (35)	474.90 > 110.10 (77)	474.90 > 227.00 (35)
Cefadroxil	Cephalosporins	9.8	+	363.91 > 158.00 (21)	363.91 > 140.10 (39)	363.91 > 68.10 (59)
Cefalexin	Cephalosporins	10.1	+	348.10 > 158.00 (20)	348.10 > 106.00 (26)	348.10 > 174.00 (14)
Cefalonium	Cephalosporins	9.7	+	458.94 > 337.00 (15)	459.00 > 152.00 (25)	459.00 > 158.00 (23)
Cephaprin	Cephalosporins	8.5	+	424.00 > 292.00 (21)	424.00 > 151.90 (33)	424.00 > 181.00 (31)
Cloxacillin	Penicillins	16.5	+	435.86 > 178.00 (33)	435.86 > 220.10 (25)	435.86 > 320.90 (25)
Danofloxacin	Quinolones	10.8	+	358.00 > 340.10 (29)	358.00 > 314.10 (25)	358.00 > 96.00 (31)
Diethylcarbamazine	Anthelmintics	7.5	+	200.09 > 127.03 (20)	200.09 > 100.00 (25)	200.09 > 72.03 (30)
Diminazene	Phenylhydrazines	8.3	+	282.01 > 119.10 (25)	282.01 > 102.10 (55)	282.01 > 135.10 (27)
Doxycycline	Tetracycline	14.8	+	445.13 > 428.00 (27)	445.13 > 320.90 (43)	445.13 > 267.00 (49)
Florfenicol amine	Amphenicols	3.3	+	248.10 > 230.00 (13)	248.10 > 130.00 (28)	248.10 > 91.00 (60)
Isometamidium	Triazene Trypanocidal agents	13.1	+	460.02 > 313.10 (27)	460.02 > 298.10 (33)	460.02 > 269.10 (71)
Lincomycin	Lincosamides	9.4	+	407.20 > 359.20 (29)	407.20 > 126.10 (31)	407.20 > 82.20 (117)
Marbofloxacin	Quinolones	9.8	+	363.00 > 320.10 (21)	363.00 > 72.10 (47)	363.00 > 345.00 (29)
Metronidazole-OH	Nitroimidazole	7	+	188.00 > 126.00 (23)	188.00 > 123.10 (19)	188.00 > 41.20 (47)
Minocycline	Tetracycline	12.3	+	457.99 > 441.10 (27)	457.99 > 283.10 (59)	457.99 > 337.00 (51)
Monoacetyl dapsone	Other	12.1	+	290.05 > 262.10 (17)	290.05 > 134.10 (19)	290.05 > 179.10 (19)
Nafcillin	Penicillins	17.2	+	414.98 > 199.1 (21)	414.98 > 171.0 (51)	414.98 > 143.0 (60)
Ofloxacin	Quinolones	10.2	+	362.10 > 261.00 (40)	362.10 > 318.00 (30)	362.10 > 221.00 (45)
Orbifloxacin	Quinolones	11.1	+	396.06 > 352.10 (27)	396.06 > 295.10 (33)	396.06 > 254.00 (41)
Oxytetracycline	Tetracycline	11.4	+	461.10 > 426.00 (30)	461.10 > 201.00 (40)	461.10 > 337.00 (40)
Penicillin V	Penicillins	16.2	+	351.00 > 114.00 (40)	351.00 > 160.00 (25)	351.00 > 229.20 (25)
Phenothiazine	Neuroleptic agents	19.6	+	198.92 > 167.10 (37)	198.92 > 166.10 (51)	198.92 > 139.10 (63)
Phthalylsulfathizole	Sulfonamides	12.4	+	403.85 > 156.10 (29)	403.85 > 149.00 (47)	403.85 > 108.00 (43)
Sarafloxacin	Quinolones	11.5	+	386.01 > 299.10 (35)	386.01 > 322.10 (31)	386.01 > 348.10 (43)
Succinylsulfathiazole	Sulfonamides	10.6	+	355.84 > 108.00 (37)	355.84 > 65.00 (81)	355.84 > 73.00 (63)
Sulfisoxazole	Sulfonamides	11.8	+	268.10 > 156.00 (20)	268.10 > 113.00 (20)	268.10 > 92.00 (35)
Tetramethrin	Pyrethrins	21.2	+	332.15 > 135.07 (35)	332.15 > 107.05 (40)	332.15 > 163.99 (30)
Tulathromycin	Macrolide	10.3	+	806.53 > 577.30 (37)	806.53 > 158.10 (53)	806.53 > 116.00 (81)

## Data Availability

All available data are contained within the article.

## Supplementary Materials

The following supporting information can be downloaded at: https://www.mdpi.com/article/10.3390/molecules27144483/s1, Table S1: Validation data for determination of the matrix effect and the recovery of 30 veterinary drugs in corn, cow, and pet feeds (*n* = 5).; Table S2: Accuracy and precision according to the difference in extraction conditions in the sample preparation optimization process (*n* = 3).

## Sample Availability

Samples of the compounds are not available from the authors.
