# Supplementary material for: Development of a Quantitative Method for Detection of Multiclass Veterinary Drugs in Feed Using Modified QuPPe Extraction and LC–MS/MS
Source: Molecules. 2022 Jul 13;27(14):4483. doi: 10.3390/molecules27144483 (PMC9318824; doi:10.3390/molecules27144483)
Supplement: Supplementary file 1 [file molecules-27-04483-s001.zip › molecules-1806382-supplementary.pdf]

Supplementary Materials: Development of a Quantitative Method for Detection of Multiclass Veterinary Drugs in Feed Using Modified QuPPe Extraction and LC-MS/MS

Sunyeong Jang [1,†], Hyungju Seo [1,†], Hojin Kim [1], Hyoyoung Kim [1], Jongsung Ahn [2], Hyunjeong Cho [1], Sunghie Hong [1], Seunghwa Lee [1,*] and Taewoong Na [1,*]

Table S1. Validation data for determination of the matrix effect and the recovery of 30 veterinary drugs in corn, cow, and pet feeds (*n* = 5).

Analyte	Matrix Effect (%)	Intra-Lab (*n* = 5)						Inter-Lab (*n* = 5)					
		Low		Middle		High		Low		Middle		High	
		Recovery (%)	RSD (%)	Recovery (%)	RSD (%)	Recovery (%)	RSD (%)	Recovery (%)	RSD (%)	Recovery (%)	RSD (%)	Recovery (%)	RSD (%)
Corn feed													
**Amphenicols (1)**													
Florfenicol amine	50.32	98.78	7.53	99.33	10.76	95.50	18.74	100.66	2.63	104.06	6.42	100.19	6.62
**Anthelmintics (1)**													
Diethylcarbamazine	8.01	77.65	6.64	97.92	5.95	76.93	5.12	92.86	23.16	103.71	7.89	91.06	21.94
**Cephalosporins (4)**													
Cefadroxil	-91.68	94.49	15.24	83.24	6.42	95.37	9.22	92.29	3.37	85.31	3.42	90.46	7.68
Cefalexin	-93.35	90.53	14.46	103.51	5.84	108.98	6.45	90.63	0.16	94.20	13.98	98.39	15.22
Cefalonium	-95.22	112.02	11.37	74.07	3.67	87.98	9.46	93.58	22.88	75.54	2.75	82.52	9.36
Cephapirin	-39.38	92.52	13.95	79.94	8.29	97.69	8.29	82.73	16.74	75.22	8.88	83.93	23.20
**Coccidiostats (1)**													
Amprolium	-82.63	73.06	9.67	94.90	13.37	102.26	9.98	92.06	27.19	101.35	9.00	104.51	3.04
**Lincosamides (1)**													
Lincomycin	40.07	87.75	6.92	85.32	11.35	71.62	1.95	99.42	16.60	96.20	15.99	87.17	25.22
**Macrolide (1)**													
Tulathromycin	-21.43	105.42	10.35	114.25	8.26	101.83	1.80	104.30	1.53	113.33	1.15	105.62	5.07
**Nitroimidazole (1)**													

Metronidazole-OH	-34.93	101.84	13.31	118.42	1.67	115.20	4.37	102.37	0.73	109.45	11.60	107.31	10.41
**Penicillins (3)**													
Cloxacillin	27.70	99.00	7.40	95.51	17.89	100.40	7.83	102.51	4.84	100.58	7.12	97.75	3.83
Nafcillin	10.94	96.11	14.82	111.40	4.14	83.55	2.26	101.73	7.81	109.03	3.08	89.37	9.20
Penicillin V	-24.91	106.33	16.71	104.22	3.64	113.35	6.59	108.81	3.22	102.67	2.14	100.68	17.80
**Phenylhydrazines (1)**													
Diminazene	31.12	112.26	5.47	78.25	14.45	82.91	11.15	95.03	25.65	77.70	1.00	78.65	7.66
**Polypeptides (1)**													
Bacitracin	-94.02	95.05	12.43	108.50	3.84	107.82	9.20	94.77	0.42	100.38	11.44	102.78	6.94
**Pyrethrins (1)**													
Tetramethrin	-6.55	105.44	16.78	101.58	6.22	99.89	4.06	105.72	0.37	106.25	6.22	104.51	6.25
**Quinolones (5)**													
Danofloxacin	57.31	105.50	8.58	101.50	9.53	98.55	4.58	95.25	15.23	91.01	16.30	86.38	19.93
Marbofloxacin	39.63	105.82	9.74	102.27	4.55	108.89	11.80	94.16	17.52	91.48	16.68	93.76	22.83
Ofloxacin	43.98	73.70	15.04	106.09	3.21	104.78	8.47	78.88	9.29	95.91	15.01	93.21	17.55
Orbifloxacin	-99.21	105.47	13.65	108.29	3.38	110.31	6.43	103.77	2.32	104.87	4.61	105.09	7.02
Sarafloxacin	-23.92	89.66	8.10	78.14	9.15	82.76	7.30	83.78	9.93	76.43	3.17	76.69	11.19
**Sulfonamides (3)**													
Phthalylsulfathiazole	-34.16	96.98	6.76	84.88	6.56	109.06	5.57	93.16	5.80	85.88	1.64	98.14	15.74
Succinylsulfathiazole	-11.39	115.84	6.14	93.33	8.31	91.71	9.90	110.04	7.45	94.50	1.74	96.05	6.39
Sulfisoxazole	35.43	106.93	7.89	112.44	7.23	108.70	8.12	109.16	2.89	110.79	2.11	108.51	0.25
**Tetracycline (3)**													
Doxycycline	-40.28	115.40	7.12	88.76	16.58	95.54	10.91	102.98	17.06	85.14	6.02	85.38	16.84
Minocycline	-15.39	102.86	9.07	115.85	2.85	109.30	3.57	95.53	10.85	98.81	24.40	93.68	23.58
Oxytetracycline	-35.59	105.64	12.52	104.87	14.64	93.63	6.27	93.20	18.88	90.19	23.03	83.38	17.39
**Neuroleptic agents (1)**													
Phenothiazine	-43.13	91.72	8.86	81.83	13.77	104.09	4.29	99.67	11.27	94.93	19.51	106.40	3.06
**Triazene trypanocidal agents (1)**													
Isometamidium	11.53	89.73	8.59	107.28	4.15	100.89	10.85	82.33	12.72	90.42	26.38	86.61	23.32
**Other (1)**													
Monoacetyl dapsone	-46.75	102.09	17.32	114.43	6.57	115.71	4.75	105.81	4.97	112.58	2.33	111.74	5.02

							Cow feed						
**Amphenicols (1)**													
Florfenicol amine	99.13	107.63	7.16	89.33	13.16	79.55	3.58	105.45	2.92	98.29	12.89	93.50	21.10
**Anthelmintics (1)**													
Diethylcarbamazine	8.89	110.53	15.37	105.01	9.24	93.56	12.83	106.83	4.90	104.88	0.18	99.27	8.13
**Cephalosporins (4)**													
Cefadroxil	-93.87	91.01	16.37	86.40	2.79	81.41	6.97	90.49	0.82	86.63	0.38	86.42	8.20
Cefalexin	-94.81	101.63	14.92	82.56	11.54	95.94	7.98	95.95	8.38	85.61	5.03	94.69	1.87
Cefalonium	-95.10	90.74	13.01	79.46	9.16	72.45	5.36	86.24	7.39	84.92	9.09	79.40	12.38
Cephapirin	-48.97	107.80	3.69	97.26	11.43	108.29	9.09	92.54	23.33	88.48	14.04	95.35	19.19
**Coccidiostats (1)**													
Amprolium	0.49	102.89	7.92	86.16	7.89	98.65	9.31	99.73	4.48	94.82	12.92	102.18	4.89
**Lincosamides (1)**													
Lincomycin	4.58	96.56	12.33	95.85	6.12	90.80	13.59	103.16	9.04	101.35	7.67	97.10	9.17
**Macrolide (1)**													
Tulathromycin	-46.79	111.18	1.97	97.58	4.45	101.51	11.18	105.04	8.27	99.73	3.05	103.50	2.71
**Nitroimidazole (1)**													
Metronidazole-OH	-48.37	86.28	11.16	101.28	10.18	94.54	11.66	96.59	15.09	104.47	4.31	99.35	6.85
**Penicillins (3)**													
Cloxacillin	-30.52	104.66	0.47	91.72	3.08	104.09	4.29	101.31	4.68	97.04	7.75	102.31	2.47
Nafcillin	-60.13	102.82	15.28	108.64	15.19	91.46	12.66	106.79	5.26	109.50	1.10	98.65	10.31
Penicillin V	-54.14	85.88	14.44	94.19	11.42	95.56	15.40	94.15	12.42	96.41	3.25	99.81	6.02
**Phenylhydrazines (1)**													
Diminazene	1.32	107.80	3.69	82.57	13.84	92.47	14.95	92.41	23.56	80.33	3.94	83.28	15.62
**Polypeptides (1)**													
Bacitracin	-94.59	95.79	12.99	80.19	7.27	104.76	8.89	84.63	18.65	80.09	0.19	94.32	15.66
**Pyrethrins (1)**													
Tetramethrin	-2.49	99.76	0.83	98.30	3.72	94.85	15.31	97.30	3.58	97.93	0.54	97.05	3.21
**Quinolones (5)**													
Danofloxacin	5.82	101.14	8.91	86.91	11.90	79.72	5.04	86.38	24.17	81.14	10.06	74.97	8.97
Marbofloxacin	2.92	95.87	7.72	94.78	9.73	76.52	1.94	86.28	15.72	85.40	15.54	73.41	6.00
Ofloxacin	9.43	86.97	7.94	91.46	12.56	72.39	4.22	79.13	14.02	85.18	10.44	72.91	1.01

Orbifloxacin	3.83	90.99	11.65	93.03	12.99	85.90	10.02	86.62	7.13	90.86	3.39	92.86	10.59
Sarafloxacin	-49.99	98.07	13.56	79.88	8.90	88.50	14.37	88.92	14.56	76.56	6.14	81.30	12.52
**Sulfonamides (3)**													
Phthalylsulfathiazole	-46.59	84.18	6.59	93.53	5.65	91.87	7.47	81.15	5.28	87.10	10.44	89.78	3.29
Succinylsulfathiazole	-48.55	95.70	4.90	95.57	11.96	98.28	12.19	93.81	2.85	95.11	0.69	97.94	0.50
Sulfisoxazole	3.69	103.06	4.91	98.06	5.73	95.30	15.44	104.44	1.87	100.90	3.98	102.12	9.44
**Tetracycline (3)**													
Doxycycline	-60.66	98.59	0.93	98.71	9.63	91.39	15.80	88.06	16.91	88.77	15.84	82.14	15.93
Minocycline	-65.71	103.58	8.36	100.85	8.51	88.32	10.15	90.47	20.49	87.34	21.88	80.91	12.96
Oxytetracycline	-55.38	92.68	5.29	87.51	2.06	97.19	15.86	83.94	14.73	82.34	8.88	88.53	13.83
**Neuroleptic agents (1)**													
Phenothiazine	-44.24	87.87	7.23	89.79	6.79	79.40	6.82	94.87	10.43	98.87	12.99	93.32	21.09
**Triazene trypanocidal agents (1)**													
Isometamidium	-5.00	92.15	3.01	90.68	9.22	82.28	6.42	81.74	18.02	82.01	14.95	78.03	7.71
**Other (1)**													
Monoacetyl dapsone	-52.87	105.60	4.62	98.94	10.82	94.28	12.75	102.92	3.68	101.03	2.92	98.01	5.38
							Pet feed						
**Amphenicols (1)**													
Florfenicol amine	76.18	104.78	3.89	110.20	2.75	90.51	10.67	95.39	13.92	101.46	12.18	94.76	6.34
**Anthelmintics (1)**													
Diethylcarbamazine	91.49	103.07	14.31	96.43	14.21	95.54	13.82	91.38	18.10	96.70	0.39	95.35	0.28
**Cephalosporins (4)**													
Cefadroxil	-87.47	94.54	3.23	80.39	7.84	76.75	4.79	84.28	17.23	78.44	3.52	75.87	1.65
Cefalexin	-90.60	95.90	8.17	72.60	4.75	71.01	4.57	88.01	12.69	76.77	7.68	76.23	9.68
Cefalonium	-94.30	100.47	9.47	98.01	10.97	70.79	1.80	86.49	22.87	85.85	20.03	70.50	0.58
Cephapirin	-15.28	83.48	10.46	96.37	12.13	101.23	1.23	79.60	6.90	86.23	16.64	88.63	20.11
**Coccidiostats (1)**													
Amprolium	-77.81	70.75	9.62	103.99	8.36	72.10	3.49	82.31	19.85	101.40	3.62	84.27	20.42
**Lincosamides (1)**													
Lincomycin	82.95	97.24	10.59	93.48	4.83	89.22	17.69	97.70	0.66	96.23	4.03	93.79	6.89
**Macrolide (1)**													

Tulathromycin	-1.98	104.82	2.27	82.43	1.44	91.92	12.23	98.57	8.97	91.41	13.89	93.95	3.06
**Nitroimidazole (1)**													
Metronidazole-OH	-0.15	103.84	13.07	87.10	7.42	86.34	14.68	96.64	10.54	90.09	4.69	89.92	5.63
**Penicillins (3)**													
Cloxacillin	89.12	99.94	9.75	90.84	14.45	79.12	2.83	101.01	1.50	94.54	5.53	85.16	10.02
Nafcillin	49.81	107.64	8.41	84.84	11.39	100.17	6.71	107.65	0.01	94.62	14.61	97.12	4.45
Penicillin V	4.29	101.91	10.58	89.50	13.76	107.93	4.82	103.49	2.15	96.79	10.64	102.18	7.96
**Phenylhydrazines (1)**													
Diminazene	82.58	99.14	4.51	78.71	5.42	93.51	8.12	94.55	6.87	81.22	4.36	83.39	17.17
**Polypeptides (1)**													
Bacitracin	-92.38	92.60	6.07	85.18	7.38	101.14	10.01	85.73	11.34	80.51	8.20	89.75	17.96
**Pyrethrins (1)**													
Tetramethrin	64.13	98.13	1.46	90.03	12.45	93.62	16.70	86.13	19.71	85.53	7.45	86.04	12.47
**Quinolones (5)**													
Danofloxacin	6.24	90.84	14.61	106.11	3.11	77.82	6.38	85.58	8.69	93.56	18.97	78.45	1.14
Marbofloxacin	99.89	100.38	0.71	90.26	11.77	76.81	5.20	89.22	17.70	86.03	6.95	78.19	2.50
Ofloxacin	-98.89	108.54	9.11	87.27	8.48	74.45	3.01	96.20	18.14	85.78	2.46	78.93	8.02
Orbifloxacin	-98.95	94.79	9.02	79.18	3.35	76.77	3.06	92.50	3.51	86.87	12.51	84.66	13.18
Sarafloxacin	3.66	86.38	13.64	77.59	3.08	104.76	10.48	80.78	9.81	77.06	0.97	89.76	23.64
**Sulfonamides (3)**													
Phthalylsulfathiazole	-14.12	90.35	13.08	76.89	4.93	97.45	2.39	82.30	13.83	76.23	1.22	84.20	22.26
Succinylsulfathiazole	14.18	108.21	5.29	88.74	12.71	104.57	8.67	97.13	16.14	88.06	1.10	92.84	17.88
Sulfisoxazole	91.43	103.06	3.81	81.74	6.25	77.56	9.81	98.66	6.31	89.08	11.65	86.51	14.63
**Tetracycline (3)**													
Doxycycline	-30.68	89.64	1.77	79.69	1.61	89.14	17.30	84.87	7.96	81.51	3.16	85.12	6.68
Minocycline	7.45	105.79	2.99	84.01	2.50	83.37	14.36	93.51	18.57	80.74	5.74	81.40	3.42
Oxytetracycline	-38.31	98.85	10.01	89.12	8.27	90.60	13.29	99.84	1.40	86.16	4.87	85.90	7.74
**Neuroleptic agents (1)**													
Phenothiazine	-1.08	96.83	13.82	95.24	7.03	85.42	13.32	101.24	6.16	101.04	8.12	94.95	14.19
**Triazene trypanocidal agents (1)**													
Isometamidium	39.41	94.23	8.14	102.49	6.58	86.69	12.15	85.80	13.90	90.24	19.21	81.00	9.93

**Other (1)**												
Monoacetyl dapsone	-22.04	111.50	4.06	79.01	11.73	98.11	7.16	103.42	11.05	88.30	14.87	96.61	2.20

Table S2. Accuracy and precision according to the difference in extraction conditions in the sample preparation optimization process (*n*=3).

Compound	Extraction conditions					
	1% Formic acid in 100% MeOH		1% Acetic acid in 100% MeOH		1% Acetic acid in 70% MeOH	
	Recovery (%)	RSD (%)	Recovery (%)	RSD (%)	Recovery (%)	RSD (%)
Amprolium	99.40	4.02	89.87	2.96	94.93	1.72
Bacitracin	54.53	8.71	75.20	5.87	23.05	4.60
Cefadroxil	105.13	6.17	108.20	4.62	102.00	4.08
Cefalexin	111.03	1.24	103.93	6.03	161.27	4.94
Cefalonium	77.87	0.78	74.60	5.62	62.00	4.28
Cephapirin	88.73	4.18	76.80	2.34	93.47	0.54
Cloxacillin	95.17	17.34	92.60	15.24	115.37	3.11
Danofloxacin	101.00	8.06	93.67	9.69	91.67	3.01
Diethylcarbamazine	110.40	3.33	89.87	1.71	101.20	2.08
Diminazene	110.73	7.81	75.20	8.06	116.33	3.10
Doxycycline	91.13	13.30	108.20	5.70	94.40	10.02
Florfenicol amine	91.87	2.69	103.93	1.35	75.00	3.59
Isometamidium	94.33	5.09	74.60	3.24	100.20	15.84
Lincomycin	110.87	6.05	76.80	1.72	102.20	1.48
Marbofloxacin	98.33	3.76	92.60	3.50	94.87	1.96
Metronidazole_OH	88.13	5.41	93.67	4.37	82.47	2.83
Minocycline	24.63	9.98	95.60	0.89	22.60	13.82
Monoacetyl dapsone	106.67	6.00	104.33	4.83	109.93	6.55
Nafcillin	94.07	6.31	82.80	6.08	127.07	4.81
Ofloxacin	97.00	9.32	81.67	5.41	98.47	2.77
Orbifloxacin	105.13	6.25	99.20	4.60	101.93	7.09
Oxytetracycline	90.93	12.13	99.20	5.87	77.40	5.16
Penicillin V	82.27	11.57	86.40	6.97	94.93	13.20
Phenothiazine	105.93	8.35	84.00	4.78	23.05	0.54
Phthalylsulfathiazole	107.53	8.59	77.42	3.88	102.00	7.70
Sarafloxacine	95.00	14.27	102.27	6.45	161.27	2.99

Compound	Extraction conditions					
	1% Formic acid in 100% MeOH		1% Acetic acid in 100% MeOH		1% Acetic acid in 70% MeOH	
	Recovery (%)	RSD (%)	Recovery (%)	RSD (%)	Recovery (%)	RSD (%)
Succinylsulfathiazole	101.87	6.35	95.07	4.74	62.00	6.36
Sulfisoxazole	107.20	7.55	89.27	5.67	93.47	5.88
Tetramethrin	106.20	7.90	100.20	1.33	115.37	2.54
Tulathromycin	116.43	1.74	77.20	4.37	91.67	7.32
